# Association Between Immediate Postoperative Pulse Pressure and Three-Year All-Cause Mortality After Hip Fracture Surgery in Older Adults: A Post Hoc Analysis of a Prospective Cohort

**DOI:** 10.3390/jcm15166294

**Published:** 2026-08-14

**Authors:** Feng Gao, Ruijiang Li, Yimin Chen, Shanbin Xu, Yixiao Chen, Gang Liu, Jing Zhang, Minghui Yang, Xinbao Wu

**Affiliations:** 1 Department of Orthopedics and Traumatology, Beijing Jishuitan Hospital, Capital Medical University, 31 Xinjiekou East Road, Xicheng District, Beijing 100035, China; gaofeng@bjmu.edu.cn (F.G.); liruijiang017@163.com (R.L.);; 2 National Center for Orthopaedics, Beijing 100035, China; 3 Fourth School of Clinical Medicine, Peking University, Beijing 100035, China; 4 School of Public Health, Harbin Medical University, Harbin 150081, China

**Keywords:** hip fracture, pulse pressure, postoperative blood pressure, all-cause mortality, older adults

## Abstract

**Objectives:** The prognostic relevance of postoperative blood pressure after return to the ward is unclear. In this exploratory post hoc analysis, we investigated the associations of postoperative systolic blood pressure (SBP), diastolic blood pressure (DBP), mean arterial pressure (MAP), and pulse pressure (PP) with three-year all-cause mortality in older hip fracture patients. **Methods:** This single-centre post hoc analysis used prospectively collected data from patients aged 65 years or older who underwent hip fracture surgery under neuraxial anaesthesia between November 2018 and November 2019. Immediate postoperative blood pressure was defined as the first non-invasive cuff measurement recorded after return to the ward. PP was calculated as SBP minus DBP, and MAP as (SBP + 2 × DBP)/3. All 1052 patients were included in time-to-event analyses; patients lost to follow-up were right-censored on the date they were last known to be alive. Each blood pressure parameter was assessed in a separate adjusted Cox proportional hazards model. Restricted cubic splines were used to assess nonlinearity. **Results:** Among 865 patients with known three-year vital status, 192 had died and 673 were alive; 187 patients were censored before three years. Mean PP was lower among patients who died than among survivors (58.68 mmHg (SD 19.45) vs. 62.46 mmHg (SD 20.81); *p* = 0.019). Each 10 mmHg increase in PP was associated with a lower hazard of mortality (adjusted hazard ratio (HR) 0.914, 95% confidence interval (CI) 0.834 to 0.990; *p* = 0.033). Spline analysis showed an overall association for PP (*p* = 0.033), with no evidence of nonlinearity (*p* = 0.201). Continuous SBP, DBP, and MAP, and DBP < 60 mmHg were not independently associated with mortality. **Conclusions:** In this exploratory post hoc analysis, lower immediate postoperative PP was associated with a higher risk of three-year all-cause mortality. But PP should not be interpreted as a causal or therapeutic target.

## 1. Introduction

Hip fracture is one of the most serious fragility fractures in older adults and represents a growing global public health challenge. According to the Global Burden of Disease 2021 study, the annual number of hip fractures among people aged 55 years or older increased from approximately 5.4 million in 1990 to 14.1 million in 2021 [1]. Although age-standardised incidence rates have declined in some regions, the absolute number of hip fractures is projected to approach twice the 2018 level by 2050 because of population ageing [2]. Hip fracture is also associated with substantial long-term mortality, with reported one-year mortality rates generally ranging from 15% to 30% [3,4]. Established mortality-related factors include advanced age, male sex, higher American Society of Anesthesiologists (ASA) grade, greater comorbidity burden, impaired prefracture mobility, malnutrition, anaemia, and cognitive impairment [5,6,7]. These factors predominantly describe the patient’s preoperative health status and may not fully capture the acute physiological response to fracture, anaesthesia, blood loss, and surgical stress.

Perioperative hypotension is common in older patients undergoing hip fracture surgery and may occur intraoperatively or during the early postoperative period [8]. Potential contributors include fracture-related blood loss, pre-existing anaemia, hypovolaemia, limited cardiac reserve, anaesthesia-induced vasodilatation, and inadequate resuscitation. Previous studies have associated perioperative hypotension with postoperative complications and mortality [9]. In a prospective cohort of 528 patients, Donald et al. found that SBP < 90 mmHg between two and 24 h after surgery was independently associated with 30-day mortality, with an adjusted HR of 4.6 [10]. Paul et al. similarly reported associations between perioperative hypotension and postoperative delirium, 30-day complications, 30-day mortality, and one-year mortality [11]. Conversely, Kluger et al. did not identify intraoperative hypotension as an independent determinant of mortality or length of stay [12]. Differences in definitions, exposure duration, measurement timing, and follow-up may explain these inconsistent findings. Evidence linking blood pressure measured immediately after return to the ward with long-term survival remains limited.

Blood pressure parameters describe different aspects of cardiovascular physiology. SBP and DBP reflect the interaction of ventricular ejection, vascular resistance, and arterial compliance, whereas MAP predominantly represents average perfusion pressure. PP, defined as the difference between SBP and DBP, is influenced by stroke volume, ventricular ejection, and large-artery compliance. In stable older populations, a widened PP is commonly interpreted as a marker of arterial stiffness and has been associated with all-cause and cardiovascular mortality [13,14]. In contrast, in heart failure and acute perioperative settings, a lower PP may indicate reduced stroke volume, limited cardiac reserve, or inadequate circulating volume and has been associated with adverse outcomes [15,16]. Previous hip fracture research has examined intraoperative PP in relation to postoperative delirium, but its association with long-term mortality has not been adequately evaluated [17].

This exploratory post hoc analysis therefore investigated the associations of immediate postoperative SBP, DBP, MAP, and PP with three-year all-cause mortality in older patients undergoing hip fracture surgery. We also examined whether these associations departed from linearity. The present analysis was intended to be hypothesis-generating and to assess whether immediate postoperative blood pressure parameters, particularly PP, warrant further prospective evaluation as markers of long-term mortality risk.

## 2. Patients and Methods

### 2.1. Study Design and Participants

This was a post hoc analysis of prospectively collected data from the Beijing Jishuitan Hospital site of a multicentre, non-randomised controlled study evaluating an orthogeriatric co-management model in China [18]. The original study was registered at ClinicalTrials.gov (NCT03184896). Consecutive patients treated between November 2018 and November 2019 were eligible for the present analysis.

**Figure 1:**
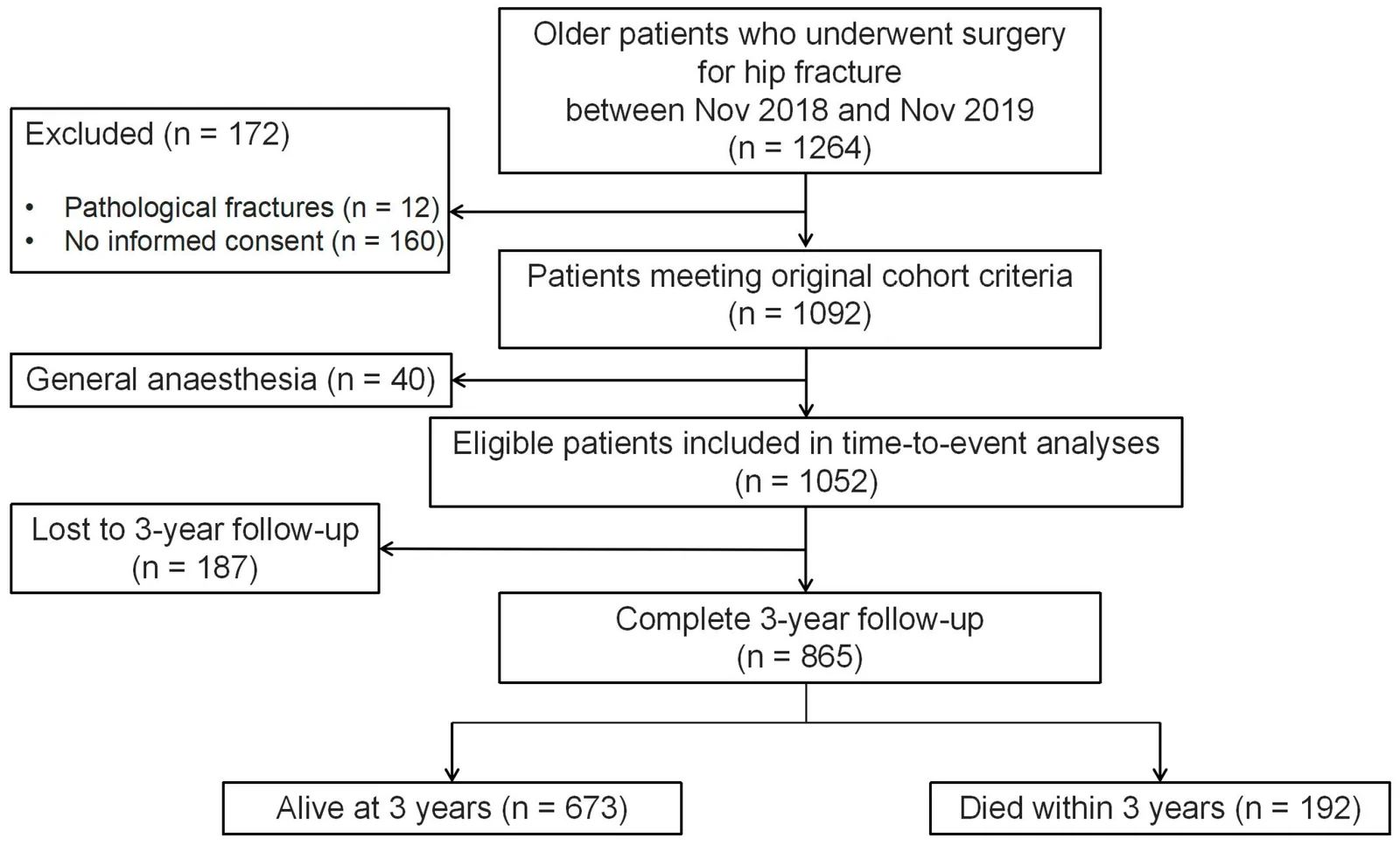
Study flowchart.

Inclusion criteria were: age 65 years or older; hip fracture confirmed by radiographs or CT, including femoral neck, intertrochanteric, or subtrochanteric fracture; admission within 21 days of injury; and operative treatment. Patients with pathological fractures, those without informed consent, and those who underwent general anaesthesia were excluded. The original study was approved by the Institutional Review Board of Peking University Health Science Center (IRB00001052-17021) and the Biomedical Ethics Committee of Beijing Jishuitan Hospital (201807-11). Written informed consent was obtained from all patients or their legally authorised representatives. The detailed patient selection and follow-up process is presented in Figure 1.

### 2.2. Perioperative Management

After diagnosis in the emergency department, patients were assessed by a multidisciplinary team comprising orthopaedic surgeons, geriatricians, emergency physicians, and anaesthetists. Preoperative assessment included routine haematological, biochemical, and coagulation tests, electrocardiography, and echocardiography when clinically indicated. Surgery was undertaken as soon as the patient was medically optimised.

All patients in this analysis underwent neuraxial anaesthesia. Undisplaced femoral neck fractures were generally treated with cannulated screw fixation, whereas displaced femoral neck fractures were treated with arthroplasty through a standard posterior approach. Stable intertrochanteric fractures (AO/OTA 31-A1) were treated with a dynamic hip screw or cephalomedullary device, and unstable fractures (AO/OTA 31-A2 or 31-A3) with a cephalomedullary device. Standard institutional protocols were used for venous thromboembolism prophylaxis and postoperative care.

### 2.3. Immediate Postoperative Blood Pressure

On return to the ward, blood pressure was measured by nursing staff using a non-invasive brachial cuff as part of routine postoperative observations. The first recorded SBP and DBP were defined as the immediate postoperative blood pressure. The exact interval between the end of surgery or anaesthesia and the ward blood pressure measurement was not systematically recorded in the parent database. Detailed information on the timing and dose of intraoperative fluid administration, transfusion, vasopressor or other vasoactive medication, and pain status at the time of measurement was also unavailable. Therefore, this measurement was interpreted as a pragmatic routine ward observation rather than a complete representation of perioperative haemodynamic exposure. PP was calculated as SBP minus DBP, and MAP as (SBP + 2 × DBP)/3. SBP < 90 mmHg was defined as low postoperative SBP, and DBP < 60 mmHg as low postoperative DBP.

### 2.4. Data Collection

Demographic and clinical data were collected prospectively using the original study protocol. Variables used in the present analysis included age, sex, body mass index (BMI), preoperative haemoglobin, ASA grade, Charlson Comorbidity Index (CCI), fracture type, prefracture mobility, diabetes mellitus, previous stroke, coronary artery disease, hypertension, and surgery within 48 h of admission. Prefracture mobility was categorised as independent, walking with an aid or assistance, or unable to walk.

### 2.5. Outcome and Follow-Up

The primary outcome was all-cause mortality within three years of surgery. Follow-up was performed by trained orthopaedic nurses using a standardised telephone protocol at approximately 30 days, one year, and three years. The date of death was recorded when applicable. Patients who remained alive at three years were administratively censored at that time. Patients who did not complete three-year follow-up were right-censored on the date they were last confirmed to be alive. Follow-up time was calculated from the date of surgery to death, last known follow-up, or three years, whichever occurred first.

### 2.6. Statistical Analysis

Continuous variables were assessed for distributional characteristics using histograms, normal probability plots, and the Shapiro–Wilk test. Normally distributed data are presented as means and standard deviations (SDs) and were compared using independent-samples t-tests. Non-normally distributed data are presented as medians and interquartile ranges and were compared using the Mann–Whitney U test. Categorical variables are presented as numbers and percentages and were compared using the Pearson chi-squared test or Fisher’s exact test, as appropriate.

Descriptive comparisons according to three-year vital status were restricted to the 865 patients whose vital status at three years was known. Time-to-event analyses included all 1052 eligible patients, with those lost to follow-up right-censored at the date they were last known to be alive. To assess the potential influence of loss to follow-up, baseline characteristics were compared between patients with known three-year vital status and those censored before three years; these comparisons are presented in Supplementary Table S1. Missing values in covariates included in the adjusted Cox models were handled using mean imputation before fitting the final multivariable Cox regression models.

Separate Cox proportional hazards regression models were used to assess the associations of immediate postoperative SBP, DBP, MAP, and PP with three-year mortality. Continuous blood pressure variables were analysed per 10 mmHg increase. Separate models were also fitted for SBP < 90 mmHg and DBP < 60 mmHg. Because SBP, DBP, MAP, and PP are mathematically related, they were not entered simultaneously into the same model. Each multivariable model was adjusted for age, sex, BMI, preoperative haemoglobin, ASA grade, CCI, fracture type, prefracture mobility, diabetes mellitus, previous stroke, coronary artery disease, hypertension, and surgery within 48 h. In the present analysis, the adjusted models included 13 prespecified adjustment domains. With 192 deaths, this corresponded to approximately 13.7 deaths per domain when the blood pressure exposure was counted. Because several covariates were categorical and may consume more than one model parameter. The proportional hazards assumption was also assessed for each Cox proportional hazards model using Schoenfeld residuals.

Potential nonlinear associations were assessed using restricted cubic splines within separate adjusted Cox models. Four knots were placed at the 5th, 35th, 65th, and 95th percentiles of each blood pressure distribution, with the median used as the reference value (HR 1.00). Wald chi-squared tests assessed the overall association and departure from linearity. All tests were two-sided, and *p* < 0.05 was considered statistically significant. No adjustment was made for multiple comparisons; the analyses were therefore interpreted as exploratory. Analyses were performed using IBM SPSS Statistics version 25.0 (IBM, Armonk, NY, USA).

## 3. Results

### 3.1. Study Cohort and Baseline Characteristics

A total of 1264 older patients who underwent surgery for hip fracture during the study period were screened. Of these, 172 patients were excluded, including 12 with pathological fractures and 160 who did not provide informed consent. Among the remaining 1092 patients, 40 who underwent surgery under general anaesthesia were excluded from the present post hoc analysis. The final study cohort therefore comprised 1052 patients, all of whom were included in the time-to-event analyses.

During the three-year follow-up period, 187 patients did not complete the final follow-up assessment and were right-censored on the date on which they were last confirmed to be alive. Three-year vital status was available for 865 patients, of whom 673 were alive and 192 had died. The study selection and follow-up process is shown in Figure 1.

Baseline characteristics of patients with known three-year vital status are shown in Table 1. Compared with survivors, patients who died were older (81.09 years (SD 7.57) vs. 79.84 years (SD 7.79); *p* = 0.048), were more often ASA grade III or IV (47.4% vs. 35.7%; *p* = 0.003), and had a different distribution of prefracture mobility (*p* = 0.039). Mean immediate postoperative SBP and PP were lower among patients who died (SBP 129.14 mmHg (SD 19.53) vs. 132.55 mmHg (SD 20.34), *p* = 0.036; PP 58.68 mmHg (SD 19.45) vs. 62.46 mmHg (SD 20.81), *p* = 0.019). DBP, MAP, the proportions with SBP < 90 mmHg or DBP < 60 mmHg, and the other recorded characteristics did not differ significantly, except that diabetes was less frequent among patients who died (20.8% vs. 28.4%; *p* = 0.042).

In addition, baseline characteristics of patients with known three-year vital status and those censored before three years are compared in Supplementary Table S1. No significant differences were observed between the two groups.

### 3.2. Cox Proportional Hazards Analyses

All 1052 patients were included in the adjusted Cox analyses, with each blood pressure parameter entered into a separate model. The results are shown in Table 2. Each 10 mmHg increase in immediate postoperative PP was associated with an 8.6% lower hazard of three-year mortality (adjusted HR 0.914, 95% CI 0.834 to 0.990; *p* = 0.033). SBP < 90 mmHg was also associated with mortality (adjusted HR 3.55, 95% CI 1.12 to 11.28; *p* = 0.032). However, only 11 patients met this threshold, including three who died. The estimate was therefore statistically imprecise and should be interpreted as an exploratory finding.

The proportional hazards assumption was assessed using Schoenfeld residuals. No statistically significant evidence of violation was detected for any Cox model: SBP, *p* = 0.408; SBP < 90 mmHg, *p* = 0.411; DBP, *p* = 0.075; DBP < 60 mmHg, *p* = 0.304; MAP, *p* = 0.070; and PP, *p* = 0.064.

### 3.3. Restricted Cubic Spline Analyses

Restricted cubic spline results are summarised in Table 3. PP was associated with three-year mortality overall (*p* = 0.033), with no evidence that the association departed from linearity (*p* for nonlinearity = 0.201). This was compatible with an approximately linear inverse association over the observed range. No significant overall or nonlinear associations were identified for SBP, DBP, or MAP.

## 4. Discussion

### 4.1. Principal Findings

In this post hoc analysis of prospectively collected data, lower PP measured immediately after return to the ward was independently associated with a higher hazard of three-year all-cause mortality after hip fracture surgery. The spline analysis showed an overall association but no evidence of nonlinearity, supporting an approximately linear inverse association over the observed range. SBP < 90 mmHg was also associated with mortality, but this finding was based on only 11 exposed patients, including three deaths, and should be regarded as an exploratory result with limited interpretive value. Continuous SBP, DBP, MAP, and DBP < 60 mmHg were not independently associated with mortality.

Viewed in the context of previous studies on perioperative hypotension after hip fracture surgery, our findings shift the focus from threshold-defined hypotension to the broader haemodynamic information contained in immediate postoperative PP. Earlier studies have mainly evaluated SBP-based hypotension, intraoperative nadirs, or postoperative hypotensive episodes over a defined time window [9,10,12]. The inconsistent findings across these studies may reflect differences in exposure definition, duration of haemodynamic disturbance, anaesthetic management, vasoactive treatment, and outcome timing. By contrast, the present study assessed the first routine ward blood pressure measurement after surgery and examined PP as a continuous parameter. This measurement should not be interpreted as a cumulative measure of hypotensive burden; rather, it represents a clinically pragmatic postoperative snapshot in which the effects of cardiovascular reserve, blood loss, fluid resuscitation, vascular tone, pain, and residual anaesthesia may converge. In this setting, PP may capture a different dimension of postoperative circulatory vulnerability from SBP, DBP, or MAP alone, although its interpretation remains limited by the lack of detailed perioperative haemodynamic timing data.

### 4.2. Interpretation Within Multidisciplinary Hip Fracture Care

Within the broader framework of contemporary multidisciplinary hip fracture care, long-term mortality after hip fracture is not only determined by a single haemodynamic variable. Instead, it reflects the combined effects of frailty, comorbidity burden, nutritional reserve and rehabilitation [19]. Current guidelines and reviews emphasize that interdisciplinary care programmes are central to improving outcomes after hip fracture [20,21].

This broader interpretation is particularly important because hip fracture commonly occurs in frail older adults with limited physiological reserve. Frailty has been consistently associated with adverse outcomes after hip fracture, and recent evidence suggests that frailty may predict mortality more strongly than chronological age alone [22]. Nutritional status is another important determinant of survival, functional recovery, and independence after hip fracture [23]. These factors may interact with postoperative haemodynamics: a low PP may be more likely to occur, and may carry greater adverse prognostic meaning, in patients with limited physiological reserve, poor nutritional status, impaired mobility, anaemia, or underlying cardiovascular disease. Therefore, immediate postoperative PP should be viewed as one component of a multidimensional postoperative assessment.

### 4.3. Physiological Interpretation and Perioperative Variability of Pulse Pressure

The inverse association observed in the present study should be interpreted in the context of acute postoperative physiological stress rather than chronic vascular ageing alone. Although a widened PP in stable older populations is often considered a marker of arterial stiffness and long-term cardiovascular risk [13,14], the first postoperative ward measurement may reflect a different haemodynamic state. After hip fracture surgery, a narrow PP may indicate reduced stroke volume, limited cardiovascular reserve, inadequate circulating volume, or incomplete haemodynamic recovery after anaesthesia. This interpretation is consistent with evidence from heart failure and high-risk surgical populations, in which lower PP has been associated with reduced cardiac reserve and adverse outcomes [15,16,24]. Thus, the present finding does not contradict the established risk associated with high PP in stable populations; rather, it suggests that the prognostic meaning of PP depends strongly on the clinical context in which it is measured.

The absence of significant associations for MAP, continuous SBP, and DBP may suggest that PP captures a different haemodynamic dimension. MAP primarily reflects mean perfusion pressure, whereas PP reflects the pulsatile interaction between ventricular ejection and the arterial circulation. A patient may therefore maintain an apparently acceptable MAP despite reduced stroke volume or impaired cardiovascular reserve. However, statistical significance for PP and non-significance for the other parameters do not establish superior predictive performance. Formal comparisons of discrimination, calibration, and incremental model fit would be required before PP could be considered a better prognostic measure.

Perioperative blood loss and volume status may also contribute to the observed association. Older patients with hip fracture can sustain substantial visible and hidden blood loss before and during surgery [25,26]. Similar preoperative haemoglobin concentrations in survivors and patients who died do not establish comparable circulating volumes, because haemoglobin is a concentration measure and may remain initially unchanged during acute blood loss or subsequently be altered by fluid shifts and resuscitation [27]. Neuraxial anaesthesia may further reduce vascular tone and venous return through sympathetic blockade [28,29]. Restricting the cohort to neuraxial anaesthesia reduced between-technique heterogeneity but did not eliminate variation in block height, local anaesthetic dose, sedation, fluid management, or vasoactive treatment [30].

PP in this study was derived from a single blood pressure measurement obtained immediately after transfer to the ward. Hidden blood loss, intravenous fluids and vasoactive drugs were not comprehensively available in the parent database. Each of these factors could influence immediate postoperative PP and could also be associated with subsequent mortality through organ hypoperfusion, myocardial injury, anaemia, delayed mobilization, or medical complications. It is therefore difficult to determine whether low PP represents an independent physiological risk factor of broader haemodynamic instability. Repeated blood pressure measurements, blood pressure trajectories, and the duration of hypotension or low PP would be required to clarify whether persistent haemodynamic patterns provide stronger prognostic information than a single ward measurement.

### 4.4. Clinical Implications

A low PP may serve as a simple haemodynamic signal that prompts a broader clinical review, including assessment for occult blood loss, hypovolaemia, anaemia, inadequate resuscitation, myocardial dysfunction, or evolving medical complications. In this context, the value of PP is primarily practical and hypothesis-generating: it may help identify patients who need more careful postoperative evaluation. Immediate postoperative PP can be calculated directly from routine ward blood pressure measurements and requires no additional testing. However, the observed effect size was modest, and the present study did not evaluate discrimination, calibration, or incremental predictive value. Therefore, PP should not be considered a validated prognostic tool.

According to the AAOS guideline, the care of older adults with hip fracture requires multidimensional perioperative management, including timely surgery, blood management, analgesia, venous thromboembolism prophylaxis, tranexamic acid use when appropriate, and interdisciplinary care to reduce complications [31]. The factors influencing mortality after hip fracture are multifactorial. In contemporary orthogeriatric care, a low postoperative PP should be integrated with the overall clinical assessment and may prompt reassessment of volume status, cardiovascular reserve, pain control, medication exposure, and the early postoperative recovery plan. Whether PP improves risk stratification beyond established predictors such as age, frailty, comorbidity burden, nutritional status, prefracture mobility, and postoperative complications remains unknown. Multidisciplinary management focusing on medical optimization, rehabilitation, fall prevention, and nutritional support remains essential for improving outcomes after hip fracture [32].

### 4.5. Strengths and Limitations

This study has several strengths. The data were collected prospectively, the blood pressure timepoint was clinically pragmatic and consistently defined, all eligible patients contributed available follow-up to the time-to-event analyses, and the outcome window extended to three years. The study also compared several routinely recorded blood pressure parameters and used restricted cubic splines to explore dose–response form.

Several limitations should be considered when interpreting these findings. First, although the data were collected prospectively, the present analysis was post hoc and the hypothesis regarding immediate postoperative PP was not prespecified in the original cohort design. The findings should therefore be regarded as hypothesis-generating. Second, PP was calculated from a single blood pressure measurement obtained immediately after return to the ward. This improves clinical feasibility but does not capture postoperative blood pressure trajectories, the duration of low PP, or dynamic changes from preoperative to postoperative blood pressure. The measurement may also have been influenced by transient factors such as residual neuraxial anaesthesia, pain, timing of assessment, fluid administration, transfusion, and vasoactive therapy. Therefore, we cannot determine whether low PP represents a surrogate marker of broader postoperative haemodynamic instability. Third, several important perioperative variables were unavailable, including intraoperative hypotension duration, hidden blood loss, fluid balance, and vasopressor or other vasoactive drug administration. These factors may have substantially influenced both PP and mortality. Residual confounding cannot be excluded. In addition, although the covariates were prespecified based on clinical relevance, the number of adjusted variables relative to the 192 deaths was close to the lower acceptable range for model stability. Therefore, model overfitting cannot be fully excluded. Fourth, the study did not include direct measures of frailty, nutritional status, rehabilitation progress, postoperative complications, or cause-specific mortality, all of which are central to long-term outcomes after hip fracture. Finally, the present analysis was restricted to patients who underwent neuraxial anaesthesia. This restriction reduced heterogeneity related to anaesthetic technique and may have improved internal consistency, but it limits external validity. The findings may not be directly generalisable to patients undergoing hip fracture surgery under general anaesthesia.

Taken together, our findings suggest that immediate postoperative PP may provide a simple early signal of haemodynamic vulnerability after hip fracture surgery. Its clinical relevance should be understood as complementary rather than definitive. In contemporary orthogeriatric care, a low PP should not be interpreted in isolation, but may prompt clinicians to reassess the patient’s volume status, anaemia, cardiovascular reserve, pain control, medication exposure, and early postoperative recovery plan. Future studies should determine whether serial PP measurements, integration with frailty and nutritional assessments, and incorporation of postoperative complications improve risk stratification beyond established clinical predictors.

## 5. Conclusions

Among older patients undergoing hip fracture surgery under neuraxial anaesthesia, lower immediate postoperative PP was independently associated with a higher risk of three-year all-cause mortality, with no evidence that the association departed from linearity. Immediate postoperative PP may be a simple marker of haemodynamic vulnerability, but it should not be interpreted as a causal factor or treatment target. External validation using repeated blood pressure measurements and more comprehensive perioperative haemodynamic data is required.

## Figures and Tables

**Table 1:** Baseline characteristics of patients with known three-year vital status.

Characteristic	Survivors (*n* = 673)	Deaths (*n* = 192)	*p*-Value
Age, years	79.84 (7.79)	81.09 (7.57)	0.048
Sex, *n* (%)			0.835
Male	198 (29.4)	55 (28.6)	
Female	475 (70.6)	137 (71.4)	
BMI, kg/m^2^	22.98 (3.99)	22.90 (3.58)	0.770
Preoperative haemoglobin, g/L	115.75 (18.22)	114.27 (16.53)	0.332
Immediate postoperative SBP, mmHg	132.55 (20.34)	129.14 (19.53)	0.036
SBP < 90 mmHg, *n* (%)	8 (1.2)	3 (1.6)	0.716
Immediate postoperative DBP, mmHg	68.77 (11.61)	68.97 (11.72)	0.837
DBP < 60 mmHg, *n* (%)	154 (22.9)	35 (18.2)	0.169
Immediate postoperative MAP, mmHg	90.03 (12.18)	89.03 (12.35)	0.322
Immediate postoperative PP, mmHg	62.46 (20.81)	58.68 (19.45)	0.019
ASA grade, *n* (%)			0.003
I to II	433 (64.3)	101 (52.6)	
III to IV	240 (35.7)	91 (47.4)	
CCI, *n* (%)			0.151
0 to 1	449 (66.7)	131 (68.2)	
2 to 3	206 (30.6)	51 (26.6)	
≥4	18 (2.7)	10 (5.2)	
Fracture type, *n* (%)			0.425
Femoral neck fracture	346 (51.4)	89 (46.4)	
Intertrochanteric fracture	311 (46.2)	99 (51.6)	
Subtrochanteric fracture	16 (2.4)	4 (2.1)	
Prefracture mobility, *n* (%)			0.039
Independent	469 (69.7)	134 (69.8)	
Walking with aid or assistance	186 (27.6)	58 (30.2)	
Unable to walk	18 (2.7)	0 (0.0)	
Diabetes mellitus, *n* (%)	191 (28.4)	40 (20.8)	0.042
Previous stroke, *n* (%)	125 (18.6)	35 (18.2)	0.914
Coronary artery disease, *n* (%)	134 (19.9)	46 (24.0)	0.223
Hypertension, *n* (%)	379 (56.3)	116 (60.4)	0.311
Surgery within 48 h, *n* (%)	507 (75.3)	136 (70.8)	0.208

Values are mean (SD) unless otherwise stated. Continuous variables were compared using independent-samples *t*-tests; categorical variables were compared using Pearson chi-squared or Fisher’s exact tests, as appropriate. ASA, American Society of Anesthesiologists; BMI, body mass index; CCI, Charlson Comorbidity Index; DBP, diastolic blood pressure; MAP, mean arterial pressure; PP, pulse pressure; SBP, systolic blood pressure.

**Table 2:** Adjusted associations between immediate postoperative blood pressure parameters and three-year all-cause mortality.

Blood Pressure Parameter	The Number of Deaths per Variable	Adjusted HR	95% CI	*p*-Value
PP, per 10 mmHg increase	13.7	0.914	0.834 to 0.990	0.033
SBP, per 10 mmHg increase	13.7	0.961	0.895 to 1.041	0.305
SBP < 90 mmHg	13.7	3.55	1.12 to 11.28	0.032
DBP, per 10 mmHg increase	13.7	1.105	0.970 to 1.255	0.130
DBP < 60 mmHg	13.7	0.808	0.563 to 1.160	0.248
MAP, per 10 mmHg increase	13.7	1.020	0.904 to 1.161	0.712

Each parameter was entered into a separate multivariable Cox model adjusted for age, sex, BMI, preoperative haemoglobin, ASA grade, CCI, fracture type, prefracture mobility, diabetes mellitus, previous stroke, coronary artery disease, hypertension, and surgery within 48 h. CI, confidence interval; DBP, diastolic blood pressure; HR, hazard ratio; MAP, mean arterial pressure; PP, pulse pressure; SBP, systolic blood pressure. Missing covariate values were imputed using mean imputation before fitting the adjusted Cox models.

**Table 3:** Restricted cubic spline analyses of immediate postoperative blood pressure parameters and three-year all-cause mortality.

Blood Pressure Parameter	*p*-Value for Overall Association	*p*-Value for Nonlinearity
SBP	0.705	0.864
DBP	0.336	0.570
MAP	0.553	0.374
PP	0.033	0.201

Separate adjusted Cox models incorporating restricted cubic spline functions were constructed for each parameter. The overall *p*-value tests the combined linear and nonlinear association; the *p*-value for nonlinearity tests departure from linearity. DBP, diastolic blood pressure; MAP, mean arterial pressure; PP, pulse pressure; SBP, systolic blood pressure.

## Data Availability

The data presented in this study are available on reasonable request from the corresponding authors. The data are not publicly available due to institutional data-management policies and privacy restrictions related to human participant data.

## Supplementary Materials

The following supporting information can be downloaded at: https://www.mdpi.com/article/10.3390/jcm15166294/s1, Table S1. Baseline Characteristics of Patients With Known Three-Year Vital Status and Those Censored Before Three Years.

## Abbreviations

Abbreviation	Definition
AO/OTA	Arbeitsgemeinschaft für Osteosynthesefragen/Orthopaedic Trauma Association
ASA	American Society of Anesthesiologists
BMI	body mass index
CCI	Charlson Comorbidity Index
CI	confidence interval
CT	computed tomography
DBP	diastolic blood pressure
HR	hazard ratio
MAP	mean arterial pressure
PP	pulse pressure
SBP	systolic blood pressure
SD	standard deviation
